# When will Two Agents Agree on a Quantum Measurement Outcome? Intersubjective Agreement in QBism

**DOI:** 10.1007/s10773-024-05790-w

**Published:** 2024-10-05

**Authors:** Rüdiger Schack

**Affiliations:** Department of Mathematics, Royal Holloway, University of London, Egham, Surrey TW20 0EX UK

## Abstract

In the QBist approach to quantum mechanics, a measurement is an action an agent takes on the world external to herself. A measurement device is an extension of the agent and both measurement outcomes and their probabilities are personal to the agent. According to QBism, nothing in the quantum formalism implies that the quantum state assignments of two agents or their respective measurement outcomes need to be mutually consistent. Recently, Khrennikov has claimed that QBism’s personalist theory of quantum measurement is invalidated by Ozawa’s so-called intersubjectivity theorem. Here, following Stacey, we refute Khrennikov’s claim by showing that it is not Ozawa’s mathematical theorem but an additional assumption made by Khrennikov that QBism is incompatible with. We then address the question of intersubjective agreement in QBism more generally. Even though there is never a necessity for two agents to agree on their respective measurement outcomes, a QBist agent can strive to create conditions under which she would expect another agent’s reported measurement outcome to agree with hers. It turns out that the assumptions of Ozawa’s theorem provide an example for just such a condition.

## Introduction

The concept of measurement is at the very heart of quantum mechanics. In interpretations of quantum mechanics that regard unitary time evolution as fundamental, measurement is often seen as a problem that needs to be solved. By contrast, QBism [3–9] takes measurement as the starting point. According to QBism, a quantum measurement is an action, any action, that an agent takes on the world external to herself where the consequences of the action – the measurement outcomes – matter to her. A measurement device is conceptually an extension of the agent who takes the measurement action. The quantum formalism is a tool that any agent can use to optimize their choice of action.

QBism is an ambitious and ongoing project that aims to discover fundamental truths about the world [10, 11] and derive quantum mechanics from compelling physical principles [4, 12]. One of the main conclusions of QBism is that quantum mechanics is not representational. Even though its mathematical structure provides deep insights into the nature of reality, quantum mechanics does not give a description of nature per se, but functions as a guide to decision making. The quantum formalism does not describe nature in the absence of agents, but instead is *normative*, i.e., answers the question of how one *should* act.

QBist quantum mechanics is rooted in personalist Bayesian probability theory, first formulated by Ramsey [13] and de Finetti [14] and later developed into a full-fledged theory of decision making by Savage [15]. Personalist probability theory provides consistency criteria for an agent’s personal probability assignments that she should strive to satisfy in order to avoid bad consequences. It is important to recognize that it does not put any constraints on how one agent’s probability assignments should be related to another agent’s probability assignments.

Exactly like personalist probability theory, QBist quantum mechanics is fundamentally first-person. Compared to probability theory, the quantum formalism provides additional normative consistency criteria for how the probabilities an agent assigns to the outcomes of different quantum measurements should be related [4]. An agent should strive to satisfy these criteria in their decision making in order to avoid bad consequences. Similar to Savage’s decision theory, QBist quantum mechanics does not put any constraints on how one agent’s assignments of probabilities, quantum states or measurement operators should be related to another agent’s analogous assignments.

In decision theory, what matters to my decision making are the potential consequences for myself of my actions. In this spirit, QBism regards the outcome of a quantum measurement as personal to the agent taking the measurement action. The outcome of a measurement is thus not something objective and in principle available to anyone who cares to look. This tenet of QBism has led to an effortless resolution of recent versions of the Wigner’s Friend paradox [16]. The tenet implies that, if *you* want to learn the outcome of a measurement *I* have done, you will have to ask me what outcome I obtained. Furthermore, because of the personal nature of both probabilities and measurement outcomes, you and I need not see the same outcome when we both perform measurements on the same system.

Recently, Khrennikov has argued [17] that Ozawa’s so-called intersubjectivity theorem [18] invalidates the QBist tenet that a measurement outcome is personal to the agent performing the measurement. In his subsequent rebuttal of Khrennikov’s claim, Stacey has pointed out [19] that Khrennikov only succeeds in showing that a certain interpretation of Ozawa’s theorem is inconsistent with QBism. Since that interpretation is incompatible with QBism to start with, Khrennikov’s argument fails to challenge QBism.

Here we build on Stacey’s argument to address the question of intersubjective agreement in QBism. We start in Sec. 2 by contrasting two mathematically equivalent but conceptually very different ways of describing quantum measurements. In Sec. 3, we review Ozawa’s theorem, show how it can be interpreted consistently from a QBist perspective, identify the additional assumptions made by Khrennikov and explain why these are incompatible with QBism. Section 4 shows that, even though there is never a necessity for two agents to agree on their respective measurement outcomes, a QBist agent can strive to create conditions under which she would expect another agent’s reported measurement outcome to agree with hers. Section 4 also provides a brief comparison with a recent debate about intersubjective agreement in Relational Quantum Mechanics (RQM). The paper concludes with a brief summary.

## General Measurements

The most general *N*-outcome measurement on a *d*-dimensional quantum system can be described [20] by a set of arbitrary operators ($d\times d$ matrices) $A_1,\ldots ,A_N$ such that

1 $$\begin{aligned} \sum _x A_x^\dagger A_x=1\;. \end{aligned}$$

The $A_x$ are known as Kraus operators, and the operators $E_x=A_x^\dagger A_x$ form a POVM. The quantum formalism connects the Kraus operators $A_x$, the state vector $|\psi \rangle$ assigned to the system before the measurement, the probabilities $p_x$ for the outcomes *x*, and the conditional state vector $|\psi _x\rangle$ assigned to the system after the measurement through the formulas

2 $$\begin{aligned} p_x=\Vert A_x|\psi \rangle \Vert ^2 \end{aligned}$$

and

3 $$\begin{aligned} |\psi _x\rangle = p_x^{-1/2} A_x |\psi \rangle \;. \end{aligned}$$

This formulation is easily generalized to the case where the agent assigns density operators rather than pure states to the system. The formulation also covers measurements of standard quantum observables (von Neumann measurements): in this case the POVM elements $E_x$ are projectors on the eigenspaces of the observable, i.e.,

4 $$\begin{aligned} E_x = \Pi _x \;,\;\; \Pi _x\Pi _y=\delta _{xy}\Pi _y \;. \end{aligned}$$

In QBism, the above constitutes a complete mathematical description of a quantum measurement. When a QBist agent contemplates a measurement action on a quantum system, the agent assigns a quantum state to the system and identifies a set of potential measurement outcomes $x=1,\ldots ,N$. The agent then assigns a Kraus operator $A_x$ to each measurement outcome, with (1) as the only necessary restriction. This step obviously requires intimate knowledge of the experimental setup and familiarity with the quantum formalism. The choice of Kraus operators will depend on the agent’s prior beliefs, which will in turn be shaped by the agent’s experience as an experimenter. QBism interprets the conditions (2) and (3) on the probabilities and post-measurement states as normative requirements: The agent should strive to satisfy these conditions to avoid bad consequences.

It is worth stressing how radically different the above account of quantum measurement is compared to more mainstream interpretations. In QBism, the Kraus operators $A_x$ and the POVM elements $E_x$ are not objective states of affairs there for all to see. They are not “inherent properties” of the measurement device. Instead they are personal to the agent contemplating the measurement. The same applies to unitaries and Hamiltonians: in QBism, these are not inherent properties of a physical system but personal to the agent who is using the quantum formalism to help with her decision making [21].

Now assume a QBist agent intends to perform the same measurement twice in a row. By this we mean nothing more than that the agent assigns the same Kraus operators $A_x$ to both measurements. Let $|\psi \rangle$ be the agent’s initial state. Her probability for obtaining outcome *y* in the second measurement, given her outcome was *x* in the first measurement, is

5 $$\begin{aligned} p_{y|x} = p_x^{-1}\Vert A_yA_x|\psi \rangle \Vert ^2\;. \end{aligned}$$

If the $A_x$ are one-dimensional projectors, i.e., if $A_yA_x=\delta _{yx}A_x$, we have $p_{y|x}=\delta _{yx}$ for all $|\psi \rangle$, i.e., the agent will expect to get the same outcome in both measurements. But this is a special case. In general, the agent’s probability for getting the same outcome in both measurements will be less than 1.

Even for projective measurements, i.e., when $E_x=\Pi _x$ where the $\Pi _x$ are projectors, an agent does not in general expect a repeated measurement to give the same outcome. The condition $A_x^\dagger A_x=\Pi _x$ on the Kraus operators is equivalent to $A_x=U_x\Pi _x$ for an arbitrary unitary operator $U_x$, i.e., the Kraus operators need not be projectors in this case. The condition $E_x=\Pi _x$ by itself places no restrictions on the conditional probabilities (5).

To summarize, QBism understands quantum measurements from a strictly single-agent viewpoint. States, Kraus operators, unitaries, probabilities and measurement outcomes are personal to the agent making the measurement. Even a single agent does not generally expect to get the same outcome when repeating a measurement. While another agent contemplating a measurement on the same system should strive to use the formalism of quantum mechanics consistently, the formalism itself places no constraints on how the states or Kraus operators of the two agents should be related. Of course the two agents can communicate and exchange reports about their respective outcomes. Further below we will describe a situation where an agent believes that the reported outcomes will be identical. This changes nothing, however, about the personal nature of the outcomes.

There is an alternative way of describing a general quantum measurement, which goes back to Hellwig and Kraus [22, 23]. Here, in addition to the quantum system of interest, an *N*-dimensional system called the meter is introduced, along with a basis $\{|x\rangle \}$ ($x=1,\ldots ,N$). Before the measurement, the joint state of system and meter is $|\psi \rangle \otimes |1\rangle$. The measurement proceeds in two steps. First, system and meter are entangled through the action of a joint unitary (which in QBism expresses the agent’s personal judgment),

6 $$\begin{aligned} |\psi \rangle \otimes |1\rangle \longrightarrow U(|\psi \rangle \otimes |1\rangle ) \;. \end{aligned}$$

The right-hand side can be expanded in terms of the basis $\{|x\rangle \}$ to give

7 $$\begin{aligned} U(|\psi \rangle \otimes |1\rangle ) = \sum _x (A_x|\psi \rangle )\otimes |x\rangle \;, \end{aligned}$$

where the $A_x$ are linear operators satisfying the normalization condition (1). Finally a projective measurement is performed on the meter, which gives outcome *x* with probability $p_x$ given by (2) and leaves the system in the state $|\psi _x\rangle =p_x^{-1/2}A_x|\psi \rangle$ as before.

This way of describing a general quantum measurement is mathematically equivalent to the description in terms of Kraus operators given above but conceptually very different. From the QBist perspective, the introduction of the meter is unnecessary, unmotivated, and confusing [26]. It is unnecessary as the equivalent Kraus-operator formulation does not require the meter. It is unmotivated as in QBism a measurement device (which the meter is arguably supposed to model in some way) is simply an extension of the agent. It is confusing because it suggests that the introduction of the meter somehow clarifies the measurement process, where in reality it only replaces one measurement (of the system) by another measurement (of the meter).

## Ozawa’s Theorem

We will now describe Ozawa’s theorem, presented as a purely mathematical theorem. Following this we will discuss possible interpretations. We present a slightly simplified version of the theorem, effectively following Khrennikov [17].

Our version of the theorem assumes a tensor product of three finite-dimensional Hilbert spaces, corresponding to a system of interest and two meters. The meters are assumed to have fixed initial states $|\xi _1\rangle$ and $|\xi _2\rangle$. If the initial state of the system is $|\psi \rangle$, a direct projective measurement on the system will give outcome *x* ($x=1,\ldots ,N$) with probability

8 $$\begin{aligned} \pi _x=\Vert \Pi _x|\psi \rangle \Vert ^2\;, \end{aligned}$$

where the Kraus operators $\Pi _x$ are projectors. Alternatively, one could first entangle the three systems through a joint unitary *U* and then perform measurements on the two meters, with Kraus operators given by projectors $P^{(1)}_x$ and $P^{(2)}_x$, respectively. In this case, the outcome probabilities for the meter measurements are

9 $$\begin{aligned} p^{(1)}_x=\Vert (I\otimes P^{(1)}_x\otimes I)|\Psi \rangle \Vert ^2\;,\;\; p^{(2)}_x=\Vert (I\otimes I\otimes P^{(2)}_x)|\Psi \rangle \Vert ^2 \;, \end{aligned}$$

where *I* is the identity and

10 $$\begin{aligned} |\Psi \rangle =U\left( |\psi \rangle \otimes |\xi _1\rangle \otimes |\xi _2\rangle \right) \end{aligned}$$

is the joint state after the unitary interaction. The joint probability for outcomes *x* and *y* for the measurements on the two meters is

11 $$\begin{aligned} p(x,y)=\Vert (I\otimes P^{(1)}_x\otimes P^{(2)}_y)|\Psi \rangle \Vert ^2\;. \end{aligned}$$

Notice that the probabilities $p^{(1)}_x$, $p^{(2)}_x$ and *p*(*x*, *y*) refer to the case that only the two measurements on the meter are carried out.

We now impose the following condition on the otherwise arbitrary unitary *U*, projectors $\Pi _x$, $P^{(1)}_x$, $P^{(2)}_x$, and initial meter states $|\xi _1\rangle$, $|\xi _2\rangle$. We assume that, for all initial system states $|\psi \rangle$, we have

12 $$\begin{aligned} \pi _x=p^{(1)}_x=p^{(2)}_x\;,\;\;x=1,\ldots ,N\;. \end{aligned}$$

Ozawa called this condition “probability reproducibility” and proved that it implies that the measurements of the two meters have the same outcome with probability 1, or

13 $$\begin{aligned} p(x,y) = 0 \;\; \text{ unless } \;\; x=y\;. \end{aligned}$$

At first sight this is a surprising result. Why would equality of probabilities entail equality of outcomes? After all, if you roll two fair dice, most of the time the outcomes won’t match, even though the probabilities for the individual outcomes are the same.

The key to understanding Ozawa’s result lies in the assumption that equality of probabilities holds for all initial states $|\psi \rangle$. The essence of the theorem can be easily grasped by considering the special case that the projectors $\Pi _x$, $P^{(1)}_x$ and $P^{(2)}_x$ are one-dimensional (the generalization to arbitrary projectors is straightforward). We can thus write

14 $$\begin{aligned} \Pi _x=|x\rangle \langle x|\;,\;\;P^{(1)}_x=|\phi ^{(1)}_x\rangle \langle \phi ^{(1)}_x|\;,\;\;P^{(2)}_x=|\phi ^{(2)}_x\rangle \langle \phi ^{(2)}_x|\;. \end{aligned}$$

Now let *x* be any outcome and let the initial system state be $|\psi \rangle = |x\rangle$. Then $\pi _x=1$ and thus we must also have that $p^{(1)}_x=p^{(2)}_x=1$. It follows that the total state after the unitary interaction is

15 $$\begin{aligned} U\left( |x\rangle \otimes |\xi _1\rangle \otimes |\xi _2\rangle \right) = |\psi _x\rangle \otimes |\phi ^{(1)}_x\rangle \otimes |\phi ^{(2)}_x\rangle \end{aligned}$$

where $|\psi _x\rangle$ is a state vector. This holds for any *x*. Hence, for an arbitrary initial state $|\psi \rangle =\sum _x c_x |x\rangle$, the total state after the unitary interaction is

16 $$\begin{aligned} U\left( |\psi \rangle \otimes |\xi _1\rangle \otimes |\xi _2\rangle \right) = \sum _x c_x |\psi _x\rangle \otimes |\phi ^{(1)}_x\rangle \otimes |\phi ^{(2)}_x\rangle \;, \end{aligned}$$

which implies directly that $p(x,y)=0$ unless $x=y$.

As a mathematical result, Ozawa’s theorem says nothing about intersubjectivity or different observers. To arrive at their interpretation, both Ozawa and Khrennikov have to make the additional assumption that their scenario – two meters interacting with a system followed by measurements on the meters – describes two different observers measuring the same system observable. We will now show that it is this additional assumption, not the mathematical theorem, that QBism is incompatible with.

Our argument is simple. According to QBism, the quantum formalism should be viewed as a decision-theoretic tool which can be used by any agent. But when an agent uses the formalism, quantum states, unitaries, measurement operators, and measurement outcomes are all personal to that agent. Hence, from a QBist perspective, Ozawa’s theorem is about measurements that *a single agent*, say, Alice, contemplates performing on a system and two meters. Given the assumptions of the theorem, Alice expects *her* outcomes of *her* measurements on the two meters to coincide. There is no other agent in the picture. By construction, QBism is consistent with this interpretation of Ozawa’s theorem. On the other hand, the assumption that the theorem is about the measurement results of two different observers violates QBism’s key tenet that the quantum formalism should be viewed as a single-agent theory.

The above argument refutes Khrennikov’s claim that Ozawa’s theorem uncovers an inconsistency in QBism. It remains silent, however, about what QBism has to say about intersubjective agreement. We turn to this question in the following section.

## Intersubjective Agreement in QBism

A possible approach to the question of agreement between two agents – let’s call them again Alice and Bob – is for Alice to treat Bob as a physical system and to assign a quantum state to him. There is nothing wrong in principle with this and, as a thought experiment, it has led to important insights. Examples include the QBist “Copernican principle” [4] and Wigner’s friend [16, 27].

On the other hand, when Alice is not considering thought experiments but uses the quantum formalism for guidance, e.g., on how to conduct an experiment in a lab, she will not assign quantum states to other agents. As a matter of fact, physicists do not normally assign quantum states even to measurement devices and meters, since assigning a quantum state entails a whole mesh of beliefs about various measurements that could in principle be performed – a mesh of beliefs that physicists almost never hold in a typical experimental situation.

Similarly, physicists rarely if ever have the extraordinarily fine-grained beliefs entailed by assigning a unitary operator to the interaction between a system and a measurement device. Since Ozawa interprets the two meters featuring in his theorem as measurement devices, the scenario on which his theorem is based must therefore be regarded as a thought experiment. The unitary *U* assigned to the joint evolution of system and meters is a purely conceptual object, not an operator that a physicist in a lab would assign and use for guidance.

So how should Alice, our experimenter in a lab, approach a situation in which she and her colleague Bob do measurements on the same system? As we established earlier, once the QBist first-person perspective is adopted, the quantum formalism does not lead to an automatic agreement between agents. Instead, agents have to actively create conditions in which they *expect* mutual agreement. Intersubjective agreement is not an automatic consequence of the quantum formalism, but a goal that agents might strive for.

In practice, Alice would talk to Bob, examine his measurement device, and ask him what operators, states and probabilities he has assigned. She would check that his assignments satisfy the constraints of the quantum formalism, take into account his experimental expertise, and then write down her own (personal) Bayesian probabilities for the outcomes that she expects Bob to report to her. Alice would then perform her measurement, wait for Bob to make his measurement, and finally ask him what outcome he obtained.

It is important to stress a number of points here. Alice’s questions to Bob should be regarded as actions Alice takes on Bob to elicit answers from him. These answers do not exist prior to the action; they are created through the action. Furthermore, whether or not Alice expects Bob’s outcomes to agree with hers is entirely contingent. There are many common scenarios where she does not expect agreement. For instance, she might come to the conclusion that Bob’s expertise as an experimenter is insufficient.

We will now see how Ozawa’s theorem can be modified to provide a simple condition under which Alice should expect agreement with Bob. As explained at the end of Section 2, Ozawa’s scenario involving meters that interact unitarily with the system is, from a QBist perspective, unmotivated, unnecessary, and confusing. We will instead consider a scenario where both Alice and Bob make direct measurements on the same system. We therefore need to adapt Ozawa’s assumption of equal probabilities to our scenario. To make this concrete, assume that Alice contemplates a measurement on a system and that her Kraus operators are

17 $$\begin{aligned} A_x=|\chi _x\rangle \langle \chi _x|\;,\;\; x=1,\ldots ,N\;,\;\; \langle \chi _x|\chi _y\rangle =\delta _{xy} \;.\ \end{aligned}$$

If her initial system state is $|\psi \rangle$, her probability for outcome *x* is $|\langle \chi _x|\psi \rangle |^2$, and her post-measurement state, given outcome *y*, is $|\chi _y\rangle$.

Following Alice’s measurement, Bob also performs an *N*-outcome measurement on the same system. In analogy to the premise of Ozawa’s theorem, we assume that, for any $|\psi \rangle$, Alice’s probability for Bob reporting outcome *x* is equal to her probability for getting outcome *x* in her own measurement, i.e., is given by

18 $$\begin{aligned} \textrm{prob}(\text {Bob reports outcome { x}})=|\langle \chi _x|\psi \rangle |^2 \;. \end{aligned}$$

Now suppose that Alice gets outcome *y* as a result of her measurement action. Her post-measurement state is $|\chi _y\rangle$. Thus, her probability for Bob reporting outcome *y* following his measurement action is $|\langle \chi _y|\chi _y\rangle |^2=1$. This means she is subjectively certain that Bob’s report will agree with her measurement outcome.

This simple scenario, which captures the essence of Ozawa’s theorem, makes clear that the assumption (18) is the end point rather than the starting point of Alice’s analysis. The probabilities (18) may or may not reflect her degrees of belief. If they do, she will have arrived at them based on a combination of factors: Bob’s record as an experimenter and as a user of quantum mechanics, her beliefs about Bob’s measurement device, Bob’s report to her of his assignments of states and operators, her judgment that he uses the quantum formalism consistently, and her own assignments of states and Kraus operators. Once Alice adopts the probabilities (18), consistency requires that she expects Bob’s report to agree with her outcome with probability 1.

It is useful to compare QBism’s response to Ozawa’s theorem with a recent parallel debate concerning intersubjectivity in RQM [1, 25]. In this debate, Cavalcanti et al. [2] respond to a claim by Lawrence et al. [24] that “extended Wigner’s friend” thought experiments show that RQM is “incompatible with quantum mechanics.” In their response, Cavalcanti et al. establish conclusively that there is, in fact, no contradiction between RQM and these thought experiments.

One reason these parallel debates are interesting is that they highlight a fundamental difference between QBism and RQM. Lawrence et al. claim (mistakenly) that the constraints RQM puts on relative facts lead to a contradiction. In other words, superficially, it looks like RQM puts too many constraints on relative facts. QBism, by contrast, is adamant that quantum mechanics puts no constraints on how the measurement outcomes of different agents are related, and Khrennikov claims (mistakenly) that Ozawa’s theorem is in contradiction to this QBist tenet. It appears that, as far as intersubjectivity is concerned, QBism and RQM start from diametrically opposite positions.

## Conclusion

Ozawa’s “intersubjectivity theorem” is based on a scenario where two meters are coupled to a system and subsequently measured. In this paper we have contrasted Ozawa’s and Khrennikov’s interpretation of this scenario with the QBist view. In Ozawa’s and Khrennikov’s interpretation, the scenario is assumed to describe two observers measuring the same observable. We have pointed out that this interpretation does not follow from the mathematical content of Ozawa’s theorem. From a QBist viewpoint, Ozawa’s theorem is about a single agent’s expectations regarding the outcomes of that agent’s measurement actions on the system and the two meters. Hence Ozawa’s theorem does not invalidate the QBist tenet that the outcomes of the agent’s measurement actions are personal to the agent. Nothing in the quantum formalism forces two agents to agree on their respective outcomes. In QBism, intersubjective agreement is not an automatic consequence of the quantum formalism, but a goal that agents might strive for. Suitably adapted, Ozawa’s theorem can provide an agent with a condition for when to expect agreement with another agent.

This work was supported by Grant 62424 from the John Templeton Foundation. The opinions expressed in this publication are those of the author and do not necessarily reflect the views of the John Templeton Foundation.

## Data Availability

No datasets were generated or analysed during the current study.

## References

[1] Adlam, E., Rovelli, C.: Information is Physical: Cross-Perspective Links in Relational Quantum Mechanics. arXiv:2203.13342 (2022)

[2] Cavalcanti, E.G., Di Biagio, A., Rovelli, C.: On the consistency of relative facts. Eur. J. Philos. Sci. **13**, 55 (2023) 38028813 10.1007/s13194-023-00551-8 PMC10665455

[3] Fuchs, C.A.: QBism, the Perimeter of Quantum Bayesianism. arXiv:1003.5209 (2010)

[4] Fuchs, C.A., Schack, R.: Quantum-Bayesian Coherence. Rev. Mod. Phys. **85**, 1693–1715 (2013). arXiv:1301.3274

[5] Fuchs, C.A., Mermin, N.D., Schack, R.: An Introduction to QBism with an Application to the Locality of Quantum Mechanics. Am. J. Phys. **82**, 749–754 (2014). arXiv:1311.5253

[6] Fuchs, C.A., Stacey, B.C.: QBism: Quantum Theory as a Hero’s Handbook. arXiv:1612.07308 (2016)

[7] Fuchs, C.A.: Notwithstanding Bohr, the Reasons for QBism. Mind and Matter **15**, 245–300 (2017). arXiv:1705.03483

[8] Fuchs, C.A.: QBism, Where Next? arXiv:2303.01446 (2023)

[9] Schack, R.: QBism: quantum mechanics is not a description of objective reality—it reveals a world of genuine free will. The Conversation (29 March 2023) (2023)

[10] Fuchs, C.A.: On Participatory Realism. In: Durham, I.T., Rickles, D. (eds.) Information and Interaction: Eddington, Wheeler, and the Limits of Knowledge, pp. 113–134. Springer, Berlin (2016). arXiv:1601.04360

[11] Schack, R.: A QBist Reads Merleau-Ponty, in Phenomenology and QBism. New Approaches to Quantum Mechanics, edited by Philipp Berghofer and Harald A. Wiltsche, p. 144–153, Routledge, New York (2024)

[12] DeBrota, J.B., Fuchs, C.A., Pienaar, J.L., Stacey, B.C.: Born’s rule as a quantum extension of Bayesian coherence. Phys. Rev. A **104**, 022207 (2021). arXiv:2012.14397

[13] Ramsey, F.P.: Truth and Probability. In: Ramsey, F.P., Braithwaite, R.B. (eds.) The Foundations of Mathematics and other Logical Essays, pp. 156–198. Harcourt, Brace and Company, New York (1931)

[14] de Finetti, B.: Probabilism: A Critical Essay on the Theory of Probability and on the Value of Science. Erkenntnis **89**, 169–223 (1989)

[15] Savage, L.J.: The Foundations of Statistics, 2nd edn. Dover, New York (1972)

[16] DeBrota, J.B., Fuchs, C.A., Schack, R.: Respecting One’s Fellow: QBism’s Analysis of Wigner’s Friend. Found. Phys. **50**, 1859–1874 (2020). arXiv:2012.14375

[17] Khrennikov, A.: Ozawa’s Intersubjectivity Theorem as objection to QBism individual agent perspective. arXiv:2301.04014 (2023)

[18] Ozawa, M.: Intersubjectivity of outcomes of quantum measurements. arXiv:1911.10893 (2019)

[19] Stacey, B.C.: Whose Probabilities? About What? A Reply to Khrennikov. arXiv:2302.09475 (2023)

[20] Nielsen, M.A., Chuang, I.L.: Quantum Computation and Quantum Information Cambridge University Press (2000)

[21] Fuchs, C.A.: Quantum Mechanics as Quantum Information (and only a little more). abridged version In Khrennikov, A. (ed.) Quantum Theory: Reconsideration of Foundations, pp. 463–543. Växjö University Press, Växjö, Sweden (2002). arXiv:quant-ph/0205039v1 (2002)

[22] Hellwig, K.E., Kraus, K.: Pure Operations and Measurements. Commun. Math. Phys. **11**, 214–220 (1969)

[23] Hellwig, K.E., Kraus, K.: Operations and Measurements. II. Commun. Math. Phys. **16**, 142–147 (1970)

[24] Lawrence, J., Markiewicz, M., Żukowski, M.: Relative facts of relational quantum mechanics are incompatible with quantum mechanics. Quantum **7**, 1015 (2023)

[25] Rovelli, C.: Relational Quantum Mechanics. Int. J. Theor. Phys. **35**, 1637 (1996)

[26] Stacey, B.C.: A Critical, But Hopefully Cordial, QBist Reply to Ballentine. arXiv:2311.06904 (2023)

[27] Wigner, E.P.: Remarks on the Mind-Body Question. In: Good, I.J. (ed.) The Scientist Speculates, pp. 284–302. William Heinemann, Ltd., London (1961) reprinted in Wigner, E.P. Symmetries and Reflections: Scientific Essays of Eugene Wigner, pp. 171–184. Ox Bow Press, Woodbridge, CT (1979)

